# Nutrition related non-communicable diseases and sugar sweetened beverage policies: a landscape analysis in Zambia

**DOI:** 10.1080/16549716.2021.1872172

**Published:** 2021-04-20

**Authors:** Mulenga M. Mukanu, Safura Abdool Karim, Karen Hofman, Agnes Erzse, Anne-Marie Thow

**Affiliations:** a Health Policy and Management Unit, University of Zambia School of Public Health, Lusaka, Zambia; b SAMRC Centre for Health Economics and Decision Science - Priority Cost Effective Lessons for Systems Strengthening (PRICELESS SA), University of the Witwatersrand, School of Public Health, Johannesburg, South Africa; c Menzies Centre for Health Policy, School of Public Health, The University of Sydney, Sydney, Australia

**Keywords:** Fiscal policies, non-communicable diseases, policy incoherence, NCD prevention policies, policy analysis, sugar reduction policy

## Abstract

**Background**: Taxation on unhealthy products is recommended as a cost-effective intervention to address the global burden of non-communicable diseases. Taxation of sugar-sweetened beverages dis-incentivize consumption of unhealthy products. Implementation of such policies is difficult in Sub-Saharan African countries, which are targets for global corporate expansion by the sugar-sweetened beverages industry.

**Objective**: To identify opportunities to strengthen policies relating to sugar-sweetened beverage taxation in Zambia, through: (1) understanding the policy landscape and political context in which policies for nutrition-related non-communicable diseases are being developed, particularly sugar-sweetened beverage taxation, and exploring the potential use of revenue arising from sugar-sweetened beverage taxation to support improved nutrition.

**Methods**: We conducted a retrospective qualitative policy analysis with a review of nutrition-related non-communicable diseases policies and key informant interviews (n = 10) with policy actors. Data were coded and analyzed data using pre-constructed matrices based on the Kingdon’s Policy Agenda Framework.

**Results**: Government responses to nutrition-related non-communicable diseases were developed in an incoherent policy environment. The health sector’s commitment to regulate sugar-sweetened beverages conflicted with the manufacturing sector’s priorities for economic growth. Increased regulation of sugar-sweetened beverages was a priority for the health sector. Economic interests sought to grow the manufacturing sector, including the food and beverage industries. Consequently, incoherent policy objectives might have contributed to the adoption of a weakened excise tax. The general public were poorly informed about nutrition-related non-communicable diseases.

**Conclusions**: The tension between the Government’s economic and public health priorities is a barrier for strengthening fiscal measures to address nutrition-related non-communicable diseases. However, this did not prevent the introduction of a differential sugar tax on sugar-sweetened beverages. Opportunities exist to strengthen the existing taxation of sugar-sweetened beverages in Zambia. These include a more inclusive consultation process for policy formulation and comprehensive monitoring of risk factors.

## Background

The Zambian government is grappling with a double burden of disease, involving communicable and non-communicable diseases, as well as challenges of high unemployment and poverty [1]. In Zambia, nutrition-related non-communicable diseases (NR-NCDs) occur, in addition to, the challenges of undernutrition and micronutrient deficiency. NR-NCDs were estimated to account for 29% of all deaths in Zambia in 2018 [2]. Latest population level data estimate that over 90% of Zambian adults do not meet the recommended five servings of fruit and/or vegetables on average per day. As a result, 24.2% of adults were found to be overweight while 7.5% were obese [3].

Evidence suggests that a nutrition transition is underway in Zambia, with people moving away from traditional foods towards a more westernized diet comprising of mainly processed and/or fried foods [4]. By 2015, sugar sweetened beverages were consumed in at least 12.8% of households in Zambia [5]. The growing and now heterogeneous soft drink market in Zambia reflects this trend too. The local market was previously dominated by Zambian Breweries and their production of Coca-Cola products [6]. However, in 2001, Varun Beverages began producing PepsiCo beverages and in subsequent years’ medium- and smaller-sized firms such as Californian Beverages entered the soft drinks market [6].

The taxation of soft drinks has been a contentious issue in Zambia for the last 20 years facing considerable opposition from the growing beverage industry. In 1998, the Zambian government chose to maintain a 25% excise tax on soft drinks amidst threats that Coca-Cola would pull out from the country [7]. However, the excise tax on soft drinks was repealed in 2015, ostensibly for economic reasons [8]. Similarly, the excise tax on alcohol reduced from 75% to 40% between 2009 and 2011, to prioritize economic growth within the broader beverage industry [9].

In 2018, the Minister of Finance announced a new excise tax on soft drinks following lobbying initiated by the health sector [10]. This announcement was supported by economic modeling showing the health impact and revenue generation a 25% SSB tax would have in Zambia [5]. However, the final policy did not follow this 25% recommendation and instead adopted a lower K0.30 per liter (USD 0.02) excise duty on non-alcoholic beverages [10]. At the time this research was conducted in 2018, the tax had not been passed as law.

The use of fiscal measures, such as SSB taxation, by government to regulate the environment for obesogenic products is bold and timely. However, there is still opportunity to strengthen the proposed SSB tax to align with evidence-based recommendations. The proposed rate of the Zambian tax is 3%. This is well below the minimum World Health Organization (WHO) recommended rate of at least 20% [11]. This rate is also lower than the rate used in research relied upon by the Ministry of Health (MoH) to propose the adoption of an SSB tax. This research, which was a local modeling study, showed that a 25% tax on SSBs could reduce SSB consumption and generate additional revenue for the Zambian government [5]. The study showed that reducing SSB consumption could significantly contribute to the reduction of premature deaths especially among young women. In addition, the proposed tax broadly applies to all non-alcoholic beverages including bottled water, and does not result in differential pricing between SSBs and non-sugary beverages. Consequently, the tax design appears to be focused more on revenue generation rather than reducing consumption of SSBs. This structure of excise taxation of soft drinks without differential rates for SSBs has been adopted as a revenue generation mechanism in many countries in Sub-Saharan Africa (SSA).

Our study aimed to identify opportunities to strengthen policies relating to sugar-sweetened beverage taxation in Zambia, through: (1) understanding the policy landscape and political context in which policies for NR-NCDs are being developed, and particularly sugar-sweetened beverage taxation, and exploring the potential use of revenue arising from SSB taxation to support improved nutrition.

## Methods

### Study design and framework

This study was part of a broader regional study aimed at analyzing the NR-NCD policy landscape and assessing the readiness to adopt SSB taxation policies in seven countries in SSA [12]. Due to the fact that Zambia was in the process of implementing an SSB tax, this study was a retrospective qualitative policy analysis. We drew on multiple frameworks in the study design. The Walt and Gilson policy analysis triangle [13] was utilized to underpin the data collection strategy and develop a data extraction matrix. In order to comprehensively map actors and their influence on the policymaking process we utilized Varvasovsky and Brugha’s approach to stakeholder analysis [14] and the Mialon et al framework [15] to systematically map corporate political activity. We also drew on Kingdon’s multiple streams approach [16] to inform the study tools and analysis. This considers the problem stream, policy stream and politics stream [16] to assess what factors lead to the opening of a ‘policy window’ which in this case, is the adoption of the SSB tax. Further detail on the study frameworks and the study methodology as it relates to this desk-based review, framework selection and application, is outlined in the study design paper [12].

### Data collection

We reviewed policies (n = 10) related to NR-NCDs as well as those specific to SSB taxation. Relevant data from the policy documents were extracted and coded using pre-determined data extraction matrices, including the policy objectives, governance and framing of the policy problem and solutions. Further detail on the documentary data collection and analysis is provided in the study design paper [12].

In addition, this study utilized interviews with key informants and stakeholders. Based on the desk review, stakeholders relevant to the adoption of the SSB tax (n = 15) were identified. Snowballing was used to identify an additional two respondents. Respondents were formally invited to participate in the study through appropriate institutional channels. The lead researcher conducted interviews (n = 10) with stakeholders from government (both health (n = 3) and economic (n = 3) sectors), a multilateral agency (n = 1), civil society organizations (n = 2) and industry (n = 1). A total of seven stakeholders (government, health (n = 2); government, economic (n = 3); government, agriculture (n = 1) and government, education (n = 1)) did not respond to the invitation (Table 1).

**Table 1.** Type of stakeholder, number of respondents and number of non-respondents by sectoral interest

Type of stakeholder	Sectoral interest of respondents who agreed to interview (n = 10)	Sectoral interest of respondents who did not agree to interview (n = 7)
National government -health	Public health (n = 2)	Government health (n = 2)
Civil society organization	Nutrition advocates (n = 4)	Government, economic/industry sector (n = 3)
National government -economic (Multi-sectoral)	Government economic sector (n = 3)	Government, agriculture sector (n = 1)
Industry	Industry (n = 1)	Government, education sector (n = 1)

The interview guide was developed based on policy theory, particularly Kingdon’s Multiple Streams Approach [16], and the findings of the desk review. The interview schedule was tailored to each respondent’s expertise. Respondents were asked about the factors leading to the adoption of the SSB tax, and opportunities and potential challenges in strengthening SSB taxation, with consideration given to the nature of the policy ‘problem’, SSB taxes as a potential ‘solution’ and the political context, and how revenue from such a tax could be used to promote access to healthy foods. All interviews were recorded and transcribed verbatim before analysis.

### Data analysis

Data analysis was iterative. First, the lead author deductively coded and thematically analyzed the interview data. We then analyzed both the documentary data and coded interview data together using Kingdon’s Multiple Streams Approach [16], with a particular focus on what factors had supported the adoption of the SSB tax in Zambia, and the way in which these had influenced the content and structure of the final taxation policy adopted. The research team reviewed the matrices and the interview data with reference to theory (see frameworks above) regarding factors known to be influential in policy making, including actors, framing and existing policy content and priorities.

### Ethical implications of the study

This study adhered to all the standards of ethical conduct of research with human subjects. All respondents voluntarily agreed to participate and provided an informed consent. Confidentiality was maintained and no risk incidence to participants was recorded. The study was approved by the ERES Converge IRB (IRB No.00005948, EWA No. 00011697), approval number 2018-Nov-021.

## Results

Kingdon’s multiple streams framework is used to present the findings [16]. We integrated data from both interviews and document review. These findings are presented under three broad categories: stream 1 presents data about the understanding of the problem of NR-NCDs and SSB taxation, a description of the current policy environment is presented in stream 2 and stream 3 gathers the findings with respect to the broader political context with respect to the major stakeholders.

### Category 1: the problem stream

This category describes the framing of the problem of NR-NCDs and SSB taxation in policy documents and the perceptions of the stakeholder respondents. It then describes the evidence gap that was identified with respect to having a better understanding of the problem.

Table 2 summarizes the content of policy documents in Zambia related to both nutrition and to NR-NCDs. Policy documents commonly framed NR-NCDs as *‘lifestyle’-*related conditions. The 7th National Development Plan (NDP) states: *‘NCD risk factors are attributable to lifestyles such as physical inactivity and unhealthy diet’* [17]. SSBs were not explicitly identified in any of the reviewed policy documents as a driver of NR-NCDs.

**Table 2.** Current policy content in relation to nutrition and NR-NCDs in Zambia by sector

Document/report	Year	Content in relation to nutrition and NR-NCDs
*Whole of government*		
Vision 2030 [28]	2006	The Government aspires to have a well-nourished population by strengthening nutrition care practices for vulnerable groups, including young children, adolescents, women in the reproductive age, and HIV/AIDS infected, and those affected by NCDs like diabetes, hypertension, coronary heart diseases, and cancer.
7^th^ National Development Plan [17]	2017–2021	Public health will be strengthened by implementing programs aimed at promoting the maintenance of clean and healthy environments and good nutrition. Efforts will be exerted towards reducing the incidences of NCDs.
*Health sector specific*		
National Health Policy [29]	2009	Particularly for NCDs, the policy objective is to prevent or delay the onset of NCDs and their related complications in order to enhance quality of life of the population.
National Health Strategic Plan [30]	2017–2021	A key shift in strategy will be to ensure that nutrition interventions are embedded in the overall plan that addresses diet-related NCDs. The Government has committed itself to establish and strengthen multisectoral plans and policies and plans for the prevention and control of NCDs.
The National Food and Nutrition Commission strategic plan [18]	2015	The strategic plan sets priority areas for government, stakeholder, donor, and partners’ spending in order to firmly position nutrition on the developmental agenda and to effectively commit predictable resources for addressing the challenges of malnutrition in Zambia (page 2). It promotes sustainable production, processing, preservation, storage, consumption and marketing of variety of food crops (especially legumes, vegetables, and fruits), fish, and livestock.
NCD Strategic Plan [19]	2013–2016	Government plans to raise the priority accorded to the prevention and control of NCDs in the national agenda through advocacy and multi-sectoral partnerships; develop and implement a national multisectoral policy and plan for the prevention and control of NCDs through multi-stakeholder engagement; and establish a National NCD Task Force to deal with the complexity of NCDs (trade, commerce and advertising, human behavior, health economics, food and agriculture systems, unhealthy commodities to children and limitation of industry self-regulation).
*Agriculture sector specific*		
National Agriculture Policy [31]	2015	Government has declared agricultural development as a priority in poverty reduction and food insecurity and it is committed to increasing the agriculture budget allocation.
*Education sector specific*		
School Health and Nutrition Policy [30]	2006	Government planned to promote and improve nutrition status of learners in order to enhance and sustain their physical, social and mental well-being; promote and maintain the health status of learners through the initiation of effective health promoting activities; provide health and nutrition education and promotion of activities at all levels of the education system.
*Economic and industry specific*		
Zambia Industrial policy [21]	2018	Government plans to make available funds that will stimulate growth in the industrial sector: The Trade and Industrial Development Fund will support growth oriented Micro Small and Medium Enterprises in high growth sectors including agro-processing, manufacturing, tourism, gemstones and infrastructure development.
2019 Budget Statement [10]	2018	Government introduced an excise duty of 30 ngwee per liter on non-alcoholic beverages. Government plans to take appropriate fiscal measures to protect local manufacturing of non-alcoholic beverages from unfair competition while discouraging import dependence.
The customs and excise (amendment) bill [32]	2018	One of the objectives of the bill is to introduce excise duty on non-alcoholic beverages

The majority of respondents saw NR-NCDs as a significant and increasing problem in Zambia. Only the industry respondent believed NR-NCDs were not a problem in Zambia, stating they are ‘*present but not pronounced*’. Generally, respondents felt that in SSA the relative food basket cost is very high, when compared to regions such as Europe and North America where food costs less and the prevalence of obesity among adults is high. There was a perception that wealthier populations are more affected by NR-NCD. One respondent explicitly linked affluence to SSB consumption stating:

“The consumers of the same drinks and the people who are in danger are the elite, the people who went to school, the middle class, they are the elite.” - Government respondent, economic

Some respondents identified reduced consumption (or *‘shunning’*) of nutritious traditional foods in favor of unhealthy food options as a major cause of NR-NCDs in Zambia. Unhealthy foods, included processed foods high in salt, fat and sugar, were perceived as *‘high status’* foods. Respondents further stated that higher consumption of unhealthy foods was also a result of the higher cost of healthy food options compared to unhealthy ones.

“With regards to nutrition we are facing quite a number of challenges. Of course, some of them have come as a result of globalization … a lot of people are moving away from eating traditional foods to eating these fast foods, food that are not that nutritious at all.” - Government respondent, health

Government (economic) respondents and nutrition advocates felt that Zambia’s reliance on a diet with limited diversity (comprising largely of a staple, breakfast meal nshima) was an under-researched problem that might be contributing to the NR-NCD epidemic. Nshima, is refined ‘breakfast’ maize meal, high in carbohydrates and is commonly consumed twice a day. An nshima-based meal typically comprises a large portion of nshima with a relatively small side of vegetables and/or protein.

Local evidence on the burden of NR-NCDs in Zambia was available from nationally representative sources (Table 3). The table shows the availability of data across different indicators and studies. The table shows that data on consumption of SSBs and other non-fruit and vegetable foods is not readily reported except in the living conditions monitoring survey.

**Table 3.** Key data sources for NR-NCDs in Zambia

Data source	**Food**				**Diet/Consumption**			**Anthropometric**		**Disease specific**	**Health services**		**Demographic**			
	Household Expenditure	Household Source	Price	Trade volumes	Fruit & vegetable	Food	SSB	BMI	Stunting and wasting	NCD prevalence	Treatment indicators	Morbidity & mortality	Age	Sex	Residence	Education
DHS								X	X				X	X	X	X
STEPS survey					X			X		X	X		X	X	X	X
MOH Annual bulletin										X	X	X				
LCMS	X	X	X		X	X	X	X	X	X			X	X	X	X
MICS								X	X				X	X	X	X
WHS					X					X			X	X	X	X
NCD Country profile										X	X	X				
UN Comtrade				X			X									
CSO monthly bulletin			X													

DHS – Demographic Health survey; LCMS – Living Condition Monitoring Survey; WHS – World Health Survey; CSO – Central Statistical Office; UN Comtrade – United Nations Commodity Trade Statistics Database

Civil society respondents and nutrition advocates believed data on food consumption habits, such as the regular consumption of breakfast meal nshima and associated disease outcomes was inadequate. Another data gap identified by respondents was the lack of information about NCD risk behaviors in different age groups and impact of policies on health. Government (economic) and civil society respondents felt that monitoring and evaluation were required to understand the outcome of the proposed SSB tax such as tracking food consumption patterns in the population. Young people were considered a key target for behavior change by all respondents.

“You know these are some of the things that they are urgent to do … What if people are getting fat because of huge intake of nshima … and these drinks have no impact but people are just being lazy and are not exercising? There are a lot of factors that can be brought together. For obesity to come in also there are so many factors that have to come together.” Nutrition advocate

### Category 2: the policy stream

Prevention and management of NR-NCDs and associated risk factors is one of the health priorities of the Government in Zambia. Sector-wide and sector-specific (health, agriculture and education) policy documents describe government strategies to address the burden of NR-NCDs (Table 2). In the 7^th^ NDP, Government plans to strengthen primary health care and public health programs to reduce the incidence of various NR-NCDs [17]. The most recently NCD Strategic Plan ran from 2013–2016 and has not been updated. The plan included strategies for addressing key gaps in the Government’s NR-NCD response, such as conflicting policy and legal frameworks, an uncoordinated multisectoral approach to NR-NCD control, low levels of public awareness of NR-NCDs and their risk factors, and the absence of a communication strategy.

In 2019, the Minister of Finance’s budget speech proposed, ‘an excise duty of 30 ngwee (3%, or USD 0.20) per liter on non-alcoholic beverages’ [10]. This tax measure was to be applied at differential rates depending on where the beverage was produced, with locally produced nonalcoholic beverages taxed at a sixth of the proposed rate for imported beverages. The initial motivation for adopting a tax on SSBs emanated from the MoH as a response to evidence on NR-NCDs and associated risk factors. However, respondents from government (health) and civil society felt that Government had no intention of using the tax for health-related purposes and had only proposed it as a revenue generation mechanism. At the time of this study, the excise tax was part of amendments to the customs and excise act, which had not yet been adopted by Parliament.

The proposed excise tax is a beverage tax that includes SSBs in its scope. It first appeared on the Government’s policy agenda in 2018. A government respondent (health) explained that evidence from a study conducted by University of Zambia help put the SSB tax on the agenda. The study helped draw attention to the association between high consumption of SSBs and NR-NCDs, and consequences of inaction versus implementing an SSB tax [5]. Evidence from this study also swayed the Ministry of Finance to support the SSB tax as it showed how much revenue would be raised from the tax.

“… the [economic modeling study] became quite a game changer for us when we showed the potential life gained and the revenue. … as much as they would have chosen the jobs, they realized … that this was actually something they could actually grab quickly. When the projections forced them to think also innovatively about sources of finance and, and I think that’s why eventually the tax never got allocated to health because it became a general tax into the general tax pool. - Government respondent, health

The Government’s policies emphasized the multisectoral approach to addressing NR-NCDs. According to the National Food and Nutrition Commission Strategic Plan, Government wants to use a multi-sectoral approach to raise additional funding and to, ‘advocate for significant increase and predictable national budgetary allocation to support food and nutrition programmes at all levels.’ [18] Government respondents across sectors felt Government was prepared with policies in place for NR-NCDs and related risk factors, and that the president’s active engagement in promoting healthy lifestyles in mainstream media was good leadership. Contrary to this, civil society felt that the current leadership and policies were insufficient because marketing of unhealthy food products remained unregulated, nutrition programs were poorly funded and the SSB tax was inadequate to curb NR-NCDs. Civil society respondents and nutrition advocates felt that despite Government’s call for multisectoral action, no one in government was willing to make hard decisions regarding NR-NCDs stating:

“… a challenge of this with the government is an inability to prioritize and I don’t think that that’s a plus. You know nobody wants to stand up and make the hard decisions because then they could be held accountable for them later.” - Civil society respondent

A lack of adequate consultation prior to policy development was also identified as a gap in government’s leadership. Industry and civil society respondents said they were not part of the policy development consultations for SSB tax. A government respondent from the economic sector agreed indicating the need for the health sector to more effectively engage with economic stakeholders on sensitive issues such as fiscal policies. They further added that without adequate consultation, policy measures like the SSB tax could be perceived to be punitive due to a lack of buy in.

“The thing is that before such taxes are put in place, as an association we highly recommend broad consultation is done with industry players. Meaning, we call out a consultative discussion which we can call upon people in the beverages sector themselves to investigate alternative solutions to sweetening of their products.” - Industry respondent

The Government had conflicting priorities between the health and economic sectors which and this has influenced NR-NCDs prevention policies. While the Government intended to regulate the production of harmful food products like SSBs, it had also committed to promoting economic growth by investing in the processed food manufacturing sector as, *‘one of the eight priority drivers of industrialization for the country’* [19,20] The health-related objectives of SSB taxation became secondary to the Government’s intention to grow the local economy. The proposed K0.30 SSB tax only applies to imported products while locally manufactured beverages are taxed at a lower rate of K0.05, irrespective of sugar content. This differential tax rate reflects the Government’s commitment to, *‘take appropriate fiscal measures to protect local manufacturing of non-alcoholic beverages from unfair competition while discouraging import dependence’* [10]. This resulted in the watering down of the proposed SSB tax and the weakening of its health impact.

Respondents were asked to potential uses for revenue arising from the adopted SSB tax. Respondents reflected that revenues from the proposed tax could finance existing health sector policy priorities for nutrition and NCD prevention including health promotion and education, regulation of the marketing of unhealthy foods, improved food labeling, and incentivizing production of healthy foods. These suggestions reflected a perception across all respondents that the population is largely unaware of causes, prevention and impact of NR-NCDs, raising awareness should be an immediate priority for Zambia. One respondent stated that a social movement, ‘from the kitchen to the streets, to the churches, to our traditional practices’ was required for NR-NCDs to be defeated because ‘nutrition-related behaviour change is dependent on both policy and health education.’ Health promotion and education aligned with Government’s objective in preventing NR-NCDs and their risk factors [19]. Another respondent identified the role of leadership from outside government in preventing NR-NCDs saying:

“We need to engage traditionally Alangizi [marriage counsellors], and then opinion makers, the political leaders, maybe the church, they have to be engaged as well.” - Government respondent, economic

Civil society respondents added that awareness campaigns would help protect the public from misinformation by manufacturers of unhealthy foods, who market their products using tags like *‘nutrition on the go’* or *‘contains real fruit.’* They observed that misinformation was widespread in the media and that manufacturers took advantage of existing weak regulations and engaged in child-directed marketing. Respondents added that low levels of awareness on NR-NCDs in the population was contributing to this misinformation.

“So look at the maheu [traditional drink made from maize meal] for example, they say its nutrition on the go so an ordinary mother out there who hears that kind of information would actually buy this food and start giving it to their child, who might even be below the age of two (as complementary feeding) which is so wrong because at the end of the day we are actually putting these children at a huge risk of obesity and non-communicable diseases in general.” - Civil Society respondent

Government and industry respondents also felt the public should be sensitized on understanding nutrition labels and making healthy choices. These respondents felt that manufacturers, played their part by correctly labeling their products. One government respondent argued that not all NCD prevention measures are Government’s responsibility and that individuals and families needed to do more to make healthy choices about their nutrition.

With the emergent of the middle class, a lot of people, families you will find that one family has more than two cars, people don’t want to go to the market … don’t want to walk to the supermarket, I don’t think that is a government issue that is our own issue … Also, diversification of diets and what we eat, I don’t think that is a government issue that is … the problem for me is more of individual and household problem than government.” Government respondent, economic

### Category 3: the stakeholder politics stream

A government respondent confirmed that the introduction of an SSB tax was initiated and championed by the MoH using local evidence on NR-NCDs in the country from University of Zambia and NFNC [5]. The MoH is the central platform around which arguments for SSB taxation coalesced. There was a strong push back to SSB taxation from the economic sector of government and from industry role-players, with one respondent stating: *‘That measure* [SSB tax] *… it was pushed by MoH and from our point of view, we fought it because we believe that, that is pushing, or contributing to the cost of doing business.’(*Government respondent, economic).

During the process of adopting the tax industry attempted to discredit evidence supporting SSB tax. Industry actors suggested that the Zambian Government bowed to international pressure for certain policy without considering the domestic context. The industry respondent asserted, *‘Usually, government in Zambia adopts foreign policy without analyzing its implication on the domestic environment. I think this move to put the tax is strongly supported by international pressure from the World Health Organization’*. A government respondent (economic) added the SSB tax should target international conglomerates as the ‘real perpetrators’, rather than local Zambian business. Respondents agreed that industry had a history of effective lobbying and used different tactics to reach key decision-makers.

“In Zambia Coca Cola [has] massive lobbying capacity and have been able to put out a lot of I think efforts to influence policy themselves in their favor. [They] sponsor certain events, high profile … you have people who write proposition papers. I think when we were trying to advocate for more taxes on tobacco and alcohol for instance, I think it was SAB miller did a presentation and sent it directly to the Minister of Finance, basically providing alternatives for funding, things like that.” - Government respondent, health

In addition, drinks manufacturers used corporate social responsibility activities (CSR) to contest the introduction of the SSB tax. The manufacturers argued that the CSR activities that sponsor benefits society. They also felt industry should be allowed to self-regulate because they undertake voluntary activities for the good of society. Civil society and government respondents felt that CSR activities were no substitute for industry taking full responsibility for the consequences of their unhealthy products.

Respondents felt that Government could use excise revenue to prioritize investments into building healthy diversified food systems based on locally available products. They argued this could be done through meaningful collaborations between key sectors such as agriculture, commerce and trade and the private sector.

Government respondents felt government sectors do not fully appreciate their interconnectedness which resulted in a misunderstanding of the intent of the SSB tax. A government respondent (health) explained that during SSB tax development, the government economic sector (along with industry) argued that the tax would lead to job losses and reduced revenue. Economic sector interviewees reiterated this assertion stating: *‘poverty levels are very high, around 60%, so if we introduce this tax to that level [the 15–20% level recommended by the World Health Organisation for public health impact], what will happen is those two, three companies which are making those drinks will close and you are going to push another 2000 direct families out of employment’*. A government respondent remarked that *‘disrupting a way of life is not something that the government embarks on.’*

A civil society respondent noted that better communication on the far-reaching consequences of NR-NCDs at all levels of society is necessary to promote policy coherence and foster multisectoral action.

“The knock-on effects are also extremely significant. And I think that’s something that we don’t do a good job … of telling the story of this problem … Essentially [comparing] every job loss to every, every family member who, who dies [of NR-NCDs] you have X number of dependents who will lose their means of support.” - Civil society respondent

## Discussion

One of our study’s objectives was to establish a comprehensive understanding of the policy landscape and political context in which policies for NR-NCDs are being developed in SSA and, to inform future policy making related to SSB taxation. Our study found that the Zambian MoH using locally generated evidence was able to get SSB on the policy agenda. However, during the adoption of the policy, opposition from industry actors led to the diluted policy announced in the 2018 national budget speech. Although the tax rate is well below the 25% recommended by WHO and the locally conducted modeling study for NCD prevention, the move by the Zambian Government is an important step in the right direction.

One learning from the Zambian experience is that taxation on SSBs will be pitched against the national imperative to keep local business competitive and profitable [17]. This study highlighted the necessity for policy measures addressing NR-NCDs in SSA to achieve the elusive ‘win-win’ balance between protecting public health and achieving economic growth. Given the high levels of unemployment and poverty in SSA, a differential SSB tax that distinguishes between international and local producers and manufacturers may be a feasible starting point to balance economic interest with public health objectives. If leveraged appropriately, SSB taxation could support innovation and further preventive efforts by providing subsidies on fruit and vegetables, or the provision of safe portable water [21]. Such measures have been shown to improve support and contribute to taxation equity [22]. A second learning is that consultation is important with respect to contested taxation policy. The MoH may have been more successful in their policy demands if the consultation process had been more diverse. The MoH’s leadership on SSB taxation was not always recognized among key stakeholders such as industry and civil society. In particular, civil society organizations who could be instrumental in advocating for a stronger tax were not adequately engaged by MoH in the policy-making process.

Our study found that framing of the causes of NR-NCDs might affect the success of efforts to prevent NR-NCDs in SSA. Respondents indirectly suggested that NR-NCDs were primarily a problem of the middle-classes, who have more affluent lifestyles. This perception that affluence is associated with consumption of unhealthy ‘fast foods’ over unrefined traditional foods, has been reported in other studies [23–25]. Addressing NR-NCDs adequately requires changing cultural norms through education and awareness in addition to fiscal measures like SSB tax. Civil society and non-health institutions including media, schools, traditional leadership and the music industry, used previously to promote social and behaviour change for HIV, should be leveraged for NR-NCD programming [26].

All stakeholders including industry supported improved public education as one of the interventions government should invest in. Although education interventions are not amongst recommended best buy interventions to curb NR-NCDs, these are required as some recent studies have shown limited public awareness of the role and benefit of fiscal policies [27]. Respondents in this study suggested education campaigns on issues such as diversification of the local diet, food labeling and the food transition away from traditional to more westernized foods. The policy environment for NCD prevention is supportive of future education-related actions, despite the present NCD Strategic Plan ending some years ago [19]. Raising public awareness and education are included in NR-NCD policy documents but have not been adequately implemented. Young people should be targeted by campaigns as evidence indicates that this age group might be unaware of policy measures like SSB tax [27]. Further research to understand young people and their food choices would also be valuable.

There are contextual factors in Zambia that impeded more aggressive strategies for preventing NR-NCDs. These include cultural perspectives on obesity, local food consumption patterns, low levels of public awareness and knowledge of NCDs and weak civil society involvement in NCD prevention. We therefore recommend a public education campaign to promote the purpose of the proposed SSB tax and adoption of a more participatory public consultation process to support the strengthening of SSB taxation.

## Limitations

Not all stakeholders who were approached to participate in this study agreed to do so. There is a ‘public health’ orientation to the qualitative data as it is currently presented due to non-participation of certain sectors approached. Industry was poorly represented in the qualitative data and government voices were limited to the health and economic sectors. This limitation was addressed by reliance on the desk-based document review which was more representative and provided insight into the positions of other stakeholder groupings in government that did not participate in interviews.

## Conclusion

The Zambian Government has proposed an excise tax on SSBs which has the potential to contribute to prevention of NR-NCDs if strengthened to achieve public health objectives. The tax was proposed by the government health sector and its adoption faced opposition from the economic sector of government, and industry representatives. Public health advocates are of the view that this tax was introduced for economic purposes because the proposed rate is well below the level required to achieve public health impact. Our findings described a tension between health and economic sectors of government which contributed to the adoption of a weaker SSB tax in Zambia. This remains a significant barrier to strengthening fiscal measures for NCD prevention.

## Paper context

Zambia’s government has adopted a tax on sugar-sweetened beverages in response to increased consumption of unhealthy foods and a growing burden of non-communicable diseases. Though there was strong political will from the Ministry of Health, conflicting government priorities on trade and economic growth negatively affected the introduction of an evidence-based and effective tax on sugar-sweetened beverages. Improving policy coherence across government sectors is needed to effectively address the epidemic of nutrition-related non-communicable diseases.
